# The effect of life stress on the public participation of rural youth in China

**DOI:** 10.1371/journal.pone.0338097

**Published:** 2025-12-15

**Authors:** Jinhua Liu, Yichi Zhang

**Affiliations:** 1 Academy of Social Governance, Southwest Petroleum University, Chengdu, Sichuan, China; 2 School of Marxism, Chengdu Normal University, Chengdu, Sichuan, China; Addis Ababa University, ETHIOPIA

## Abstract

Youth are pivotal participants in public engagement. They use public participation to promote societal change and improve living conditions, thereby coping with life stress. Against the backdrop of rural-urban disparities in geography and socio-economic development, rural youth face limited access to information and narrower channels for public participation. Moreover, scholarly exploration of life stress among rural youth remains scant. To investigate how life stress among rural youth influences their public participation behavior, this study used data from the “Chinese Social Survey” for empirical analysis, with a total sample size of 1624 individuals and an average age of 27 years. The findings indicated that lower family stress encourages rural youth to engage in mutual cooperation and collective rights protection, while higher social stress promotes the more active expression of attitudes and participation in decision-making. To enhance the public participation of rural youth, and elevate their engagement levels, recommendations include focusing on managing life stress and promoting psychological well-being, strengthening social support networks for rural youth, initiating family education programs, devising differentiated economic support policies, and intensifying advocacy for rural youth rights and public affairs.

## Introduction

### The importance of public participation in social governance

“The Party leadership’s proposals for formulating the 14th Five-Year Plan (2021–2025) for National Economic and Social Development and the Long-Range Objectives Through the Year 2035” emphasize “improving grassroots democratic consultation mechanisms to achieve positive interaction between government governance, social adjustment, and resident autonomy.” Public participation is a cornerstone of social governance, and youth represent a vital demographic within this system. Their involvement not only strengthens community cohesion but also injects dynamism and innovative capacity into social governance—particularly under China’s Rural Revitalization Strategy, which identifies youth engagement as a key driver for modernizing rural governance and boosting rural development.

### Challenges faced by rural youth

Compared with their urban counterparts, rural youth experience distinct living conditions and developmental environments. Throughout their upbringing in rural areas, they are often confronted with various stressors, including limited educational resources, restricted employment opportunities, and inadequate public services [1]. These life stressors shape their behavioral patterns and social participation. In response, many rural youth turn to public participation as a means to reshape their external social environment and improve their own living conditions [2].

Notably, rural youth have demonstrated a positive attitude and active involvement in public affairs. Surveys indicate that 87.2% of the interviewed youth anticipate becoming farmers and settling in rural areas, positioning themselves as a youthful force for rural revitalization [3]. Through engagement in rural governance, service to local communities, and contributions to agricultural development, they play a significant role in revitalizing the countryside. Many also leverage new technologies to advance rural development, embodying the spirit of Chinese youth in the new era. A growing sense of belonging and willingness to participate is also evident, with over 70% of surveyed youth expressing a longing for rural life [4].

However, rural youth still face multiple challenges in public participation, such as low integration of talent resources, insufficient professional skills, and institutional barriers. More importantly, life stress—arising from economic pressure, career development uncertainties, and identity adaptation—may profoundly influence their willingness and ability to participate. Under the macro-policy context of rural revitalization, it is essential to analyze how life stress shapes the public participation behavior of rural youth, and how such participation, in turn, affects rural social development.

Globally, studies have explored the relationship between life stress and civic engagement, yet most focus on urban or Western contexts. Within China, existing research has paid limited attention to the uniqueness of rural youth’s life stress and its connection to public participation. By integrating policy background and empirical realities, this study aims to uncover the internal relationship between life stress and public participation among rural youth, and to analyze its special significance under China’s rural revitalization strategy. Such an investigation not only enhances understanding of the behavioral logic and social adaptation of rural youth, but also provides a robust reference for policy formulation—contributing to social stability, development, and the sustainable advancement of rural revitalization.

### Definitions and significance of public participation

In Chinese political discourse and academic circles, “public participation” is frequently used. Mainstream academic viewpoints can be categorized into two types, including narrow and broad definitions of public participation. Narrow public participation emphasizes the institutionalization of public participation in legislative processes, policy formulation, and public governance. It involves public authorities using open methods to obtain information from the public, individuals, or organizations, listen to opinions, and receive feedback to influence public decision-making and governance behaviors. Broad public participation suggests that public participation and general public involvement can refer to activities where citizens attempt to influence public policies and public life. Research underscores the significance of public participation, noting that it enables governments to better understand public needs, and thereby facilitate further transformations in governmental functions [1]. Some scholars argue that interactions between government and citizens enhance citizens’ sense of agency and social responsibility, while also improving the government’s capacity for public service, thereby fostering positive interactions between government and citizens [2]. Moreover, the public participation policy plays a significant role in improving environmental governance [3].

### The role of youth in public participation

With the continuous development of China’s economy and society in recent years, an increasing number of young people have been participating in social affairs through various means.

With the enhancement of social governance, particularly at the grassroots level in China young people are participating in political life in an orderly manner and actively engaging in the practice of whole-process people’s democracy. As of June 2021, there were 23.679 million party members aged 35 and under, accounting for 24.9% of the total number of party members. Since the 18th National Congress of the Communist Party of China, the proportion of party members aged 35 and under among newly developed party members has exceeded 80%, annually. By the end of 2021, the total number of members of the Communist Youth League reached 73.715 million [5]. Young people widely participate in people’s congresses and political consultative conferences at all levels, actively performing their duties and participating in political affairs. In 2019, young representatives and members in county-level people’s congresses and political consultative conferences accounted for 10.9% and 13.7% [6], respectively. Young people actively engage in various forms of public participation, offering suggestions and fully exercising their democratic rights on issues closely related to their own interests.

However, Chinese youth, especially rural youth, as a key force for national development and national rejuvenation, are facing multi-dimensional life stress. Data from the seventh national population census show that there are about 400 million young people aged 14–35 in China, accounting for 28.4% of the total population. Since 2000, the total number of young people has gradually decreased, and the overall population structure of our country has shown an aging trend. As the core labor force, young people are under more direct stress. According to surveys, about 93% of urban youth in the country say they feel varying degrees of life stress [7].

A common way for young people to engage in public participation is through various forms of volunteer service [8]. Studies have found a positive association between the perceived responsibility and participation of China’s youth in governance, which was significantly mediated by their perceived political efficacy [9]. The primary view of much of today’s youth is that of being victims of society, rather than being a positive influence on society as a whole.

### Empowerment through participation

In fact, young people can become empowered by their participation in institutions and decisions that affect their lives, which can lead to real positive change in the community. Therefore, some scholars seek to shift the view from youth as being problems, to empowering them to enact positive social change [6]. Participation in public affairs is beneficial, not only for pooling wisdom for the government, enhancing the scientific basis of decision-making, and achieving social fairness and justice, but also for promoting the modernization of grassroots social governance systems and governance capabilities [7], which is necessary to realize the personal values of youth and generational missions [8].

### Current youth participation trends

Despite the continuous improvement in their awareness and level of public participation, overall, the level of youth participation must be enhanced. In some youth groups, concepts such as “lying flat” and “Buddha-like” culture prevail [9], which have increased their sense of “social estrangement” and “political apathy” [10], reflecting an intensified alienation between youth and society. Therefore, the Party and the government attach great importance to the public participation of youth, and have successively introduced multiple policies to guide and regulate youth participation behaviors.

### Influencing factors of public participation

Public participation is influenced by various factors. Combining Skocpol’s model of public participation in “Diminished Democracy” (social capital theory, rational choice theory, and historical institutionalism theory) [11] with revisions and additions made by domestic scholars, it is believed that youth public participation is mainly influenced by factors such as institutional environment, organizational mobilization, social relationships, and individual consciousness.

### The role of media and education

Although traditional forms of participation, such as voting and community service hold significance, digital platforms and new media have exerted an increasingly prominent role in stimulating youth participation and mobilization. Additionally, education and policy support can facilitate the active role of youth in social and political activities [12]. When policy regulations are imperfect, the channels for youth participation and the space for their interests are restricted, thus, hindering the willingness for public participation [13]. In recent years, the openness, dialogic nature, and interactivity of social media have provided a new impetus for public participation, including enhancing capabilities and expanding channels [14]. Regarding individual consciousness, public participation is driven both by “citizen passion” [15] and rational choice. Citizens believe that public participation can help them express their demands and enable them to obtain social rewards to cope with stress [16].

### Understanding life stress among youth

In the early Western studies, the Stimulus-Response Theory integrated the interaction between individual characteristics and environmental stimuli. Stress is considered a link between demand and rational coping strategies, resulting from an imbalance between individual needs and capabilities. It represents an anxious response to the interaction between environmental stimuli and individual characteristics, including factors such as individual differences and strategies for coping with stress [17]. Generally, the Stimulus-Response Theory comprehensively examines the holistic relationship between personal characteristics and external stimuli from multiple perspectives.

As different studies have been undertaken, the concept of life stress events has been defined more clearly. Hammen’s research provides a comprehensive explanation of the concept, suggesting that life stress events are occurrences in daily life that cause significant psychological and physiological changes in individuals. It asserts that life stress events are major predictors of negative emotions, leading to emotional disorders, such as anxiety and depression [18].

Early domestic research on stress mainly focused on exploratory studies in psychology or explanations of Western stress theories, applying foreign stress theories to empirically study stress issues in China. In recent years, domestic research on youth life stress has mainly focused on three areas: First, it concentrates on occupational stress and burnout under professional and work contexts, with particular emphasis on studies of young teachers. Studies have indicated that young university teachers face significant life, work, and developmental stress due to intense economic competition and increased workloads [19]. Second, some studies have focused on the mental health aspects of stress, discussing factors influencing psychological stress and coping strategies. Research suggests that with economic and social development, young adults face stress in marriage and family life that are vastly different from those of their parents’ generation. Stress related to partner selection, housing, marital expectations, and childcare constitute important components of youth life [20]. Third, scholars have also focused on specific youth groups. Some authors have studied young urban parents, proposing that they face intensified work-family conflicts, while pursuing companionship, thereby exacerbating work stress and life anxiety [21].

In summary, existing studies provide a comprehensive perspective. On one hand, they reveal the complex situations faced by contemporary youth in dealing with multiple stress, such as marriage, family, and work, which provide valuable insights for deepening the understanding of contemporary youth life conditions, formulating relevant policies, and offering effective support [22]. On the other hand, studies have also performed relatively comprehensive investigations and discussions on the definition and significance of public participation.

### Gaps in existing research and purpose of this study

Although life stress and public participation are crucial for rural youth, there remains a significant gap in understanding their relationship, particularly within the context of rural China. This study aimed to explore how life stress among rural youth influences their public participation and offers further insights and discussion for this demographic. By examining various aspects of life stress events shaping the conditions of rural youth, this study delved into the relationship between their life stress and public participation behaviors. The findings are expected to provide policymakers, social workers, and researchers with valuable insights into the quality of life and social engagement of rural youth, facilitating comprehensive development and further promoting sustainable societal progress. A better understanding of the relationship between the life stress of rural youth and their public participation behavior will aid in addressing their needs and challenges, and provide additional support for comprehensive rural reform and revitalization efforts.

### Definition of concepts

#### Definition of life stress.

Based on domestic and international research, life stress is defined as the imbalance that occurs when environmental demands do not align with individual characteristics, leading to anxiety-inducing responses.

Life stress typically manifests in daily life and can cause significant psychological and physiological changes, potentially resulting in emotional disorders such as anxiety and depression. The living environment and social background of rural youth differ from those of urban youth, necessitating a focused examination of the unique stress faced by rural youth to accurately reflect their living conditions. Apart from common stress encountered in the broader social environment, rural youth also contend with distinct life stresses stemming from rural circumstances such as limited employment opportunities, inadequate educational resources, social integration difficulties, heavy familial expectations, and substantial economic burdens [23].

Therefore, integrating various dimensions commonly used domestically and internationally—along with the living conditions of rural Chinese youth—we constructed the following dimensional system of life stress tailored to rural youth. Rural youth face three layers of stress from personal to societal levels: 1) Economic stress, which stems from limited job opportunities and lower income levels, and a mismatch between economic and consumption levels. 2) Family stress arising from intra-family relationships within the microsystem, involving parents, siblings, spouses, children, and other family members. 3) Social stress, including unique stress from the rural environment and general stress exerted by social environments on youth.

Comprehensively assessing the life stresses faced by rural youth through such dimensions assists in formulating psychological health support and intervention measures. Furthermore, in practical applications, adjustments and supplements to those dimensions can be made according to specific circumstances.

#### Definition of public participation.

Public participation is divided into narrow and broad definitions. This article primarily examines broad public participation, which not only encompasses behaviors such as accessing public, individual, or organizational information, soliciting opinions, and influencing public decision-making and governance through open pathways facilitated by public authorities for legislative power, public policy formulation, and the implementation of public governance in deciding public affairs, but also includes all activities of citizens who attempt to influence public policies and public life.

In 1969, Sherry Arnstein’s influential paper, “A Ladder of Citizen Participation” in the Journal of the American Institute of Planners had a significant impact on public participation methods and techniques, establishing a theoretical basis for making public participation a feasible and operable technology that remains widely used. She emphasized that public participation represents a redistribution of power and can reflect citizen power.

According to the power gained by citizens in the decision-making process, Arnstein divided the ladder theory of citizen participation into three levels, with eight models of citizen participation. In public participation practices, the progression from superficial participation to deep participation contained eight types of public participation, including manipulation, guidance, informing, consulting, showcasing, cooperation, authorization, and public control, which can be shown in the form of a ladder. The first level is substantive political participation, ranked from high to low as “citizen control,” “representative power,” and “partnership”; the second level is symbolic political participation, divided into “appeasement,” “consultation,” and “informing”; the third level is “non-participation,” including “indoctrination” and “manipulation”, at which point citizens cannot influence government policies [24].

#### The stress process theory.

The stress process theory suggests that an individual’s response to stress is influenced not only by the intensity of the stressor but also by mediating factors, such as social resources and coping strategies. This theory underscores the significant role of social structure, social support, and coping mechanisms in shaping stress experiences [25].

Public participation behavior represents a specific manifestation of the stress consequences within stress process theory, while the civic participation ladder theory provides a refined measurement scale for these consequences, ranging from negative to positive and from formal to substantive. stress process theory explains why life stress leads to different behavioral outcomes, while the civic participation ladder theory clearly defines “what” these outcomes are—namely, the quality and level of participation.

### Theoretical analyses and research hypotheses

In terms of stress sources, various life stress events can become sources of life stress for rural youth. Due to the uniqueness of their environment and the common stress that may arise in the broader social environment, rural youth may face specific rural life stresses, such as inadequate educational resources and limited employment opportunities. These challenges may impact their mental health, social behavior, and willingness to engage in public participation.

Existing literature highlights the relationship between life stress and public participation, suggesting that higher levels of life stress tend to strengthen an individual’s willingness to engage in public participation. Engaging in public participation is beneficial for social development, obtaining social support, and improving living conditions. However, excessive stress may lead to a decline in the mental health status of rural youth, affecting their willingness to participate in society. Rural youth may adopt various coping strategies in response to life stress, such as evasion, ignoring problems, or actively seeking solutions. These coping strategies can have significant consequences for their mental health and social behavior.

Additionally, personal traits and existing resources, such as family background, educational level, and social support networks, play a key role in how individuals cope with stress. Rural youth may turn to social support, recreational activities, or hobbies as coping mechanisms. However, the limited resources in rural areas can reduce the effectiveness of such strategies. Education, in particular, is a crucial factor influencing stress responses and coping strategies. Rural youth with higher education levels may possess better cognitive resources and coping strategies, enabling them to better manage life stress and engage in public participation behaviors, such as voting, joining youth organizations, or participating in community activities. In contrast, rural youth with lower education levels may be more likely to adopt avoidance or neglect strategies, reducing their likelihood of participation in public affairs.

Given these considerations, there are clear gaps in understanding the specific relationships between life stress, coping strategies, and public participation among rural youth, particularly in relation to how different stress sources and personal traits influence these behaviors. In order to address these gaps, this article proposes the following hypotheses:

H1: The life stress of rural youth impacts their public participation behavior.

H2: The impact of life stress from different sources on the public participation behavior of rural youth varies.

H3: There are significant differences in the influence of educational level on stress coping strategies and public participation behavior.

**Fig 1:**
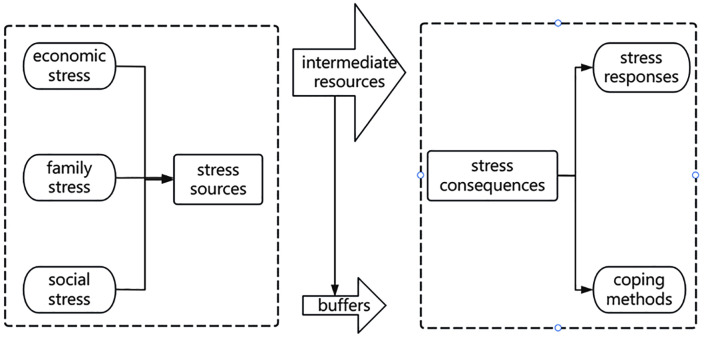
Mechanism of the stress process theory.

These hypotheses are based on the framework of the stress process theory (Fig 1). Further empirical research will verify these hypotheses using appropriate data collection and analysis methods, thereby facilitating a deeper understanding of the behavior and psychological responses of rural youth faced with life stress.

## Methods

### Data sources

This study used data from the 2021 Chinese Social Survey (CSS), a biennial longitudinal survey initiated by the Institute of Sociology at the Chinese Academy of Social Sciences in 2005. The survey utilizes household probability sampling and aims to provide longitudinal data on social changes in China during its transformation period, covering topics such as labor and employment, family dynamics, social life, and social attitudes. The survey’s data serves as a comprehensive and scientific resource for social science research and government decision-making.

The CSS2021 is the eighth wave of this survey, with a research theme focused on “Social Quality and Modernization”. It covers a wide range of topics, including family dynamics, employment, economic conditions, living standards, social security, social values and evaluation, social participation, political participation, and volunteer services. CSS2021 conducted household surveys in 592 villages/urban districts across 30 provinces, municipalities, and autonomous regions in China, gathering 10,136 valid responses.

The focus of this study is on rural areas in China. Following established research practices and theoretical frameworks, the study selected youth aged 18–35 with rural household registration as the sample. After data screening, the final sample consisted of 1,624 individuals. The sample had an average age of 27 years, with 40.58% being male and 59.42% female.

Following established research practices and theoretical frameworks, indicators were selected from the living conditions (Part D) and social and political participation (Part H) sections as measurement metrics.

This study used data from 2021 to explore life stress and public participation among rural youth for the following reasons: 1) data availability: it was relatively straightforward to obtain and organize data for 2021; 2) timeliness: a one-year time span can better reflect the current research topic and avoid the inclusion of old and irrelevant data, and 3) research cycle matching: many research projects or reports have a one-year cycle, and matching the research cycle can provide a baseline for subsequent research.

### Statistical methods and software

Literature Review Method. We conducted a comprehensive review of existing literature, thereby gaining insights into the current state and trends of research on the life stress of rural youth and public participation. Through this process, we also familiarized ourselves with the stress process theory, which forms the theoretical foundation of our study.

Statistical Analysis Method. To examine the relationship between the life stress of rural youth and their public participation behavior, we employed multivariate statistical techniques. We used statistical software, including SPSS and Stata, to conduct descriptive statistics, cross-tabulations, multiple regression analyses, and heterogeneity analyses, which collectively provide robust support for our research findings.

### Variables

#### Explanatory variable: Life stress.

The indicator selected to measure life stress was question D1a from the CSS questionnaire, asking, “In the past 12 months, which of the following aspects of life have you or your family encountered?” The question had 14 options, excluding option D1a14, and covered various life stress events such as housing conditions, children’s education expenses, caregiving burdens, family relationships, medical expenses, price levels, household income, family business status, elderly care burdens, social obligations, encounters with crime, and environmental pollution in the living environment. Such stress events were considered as constituting multiple factors of life stress for rural youth. By integrating the theoretical framework with specific questionnaire indicators, the 13 stress events were logically grouped into three distinct stress dimensions with clear discriminant validity: economic, family, and social. This categorization facilitates a more nuanced analysis of how different types of life stress differentially influence rural youth’s public participation behaviors. Economic stress encompasses indicators directly reflecting household financial conditions; family stress focuses on internal family structures and life-cycle responsibilities; while social stress points to community and public environmental issues.

These stress events were treated as encompassing multiple factors of life stress for rural youth. Through principal component factor analysis of their internal structure, new factors summarizing multiple specific indicators were extracted from the data. Each indicator of new factors was treated as a binary variable (0 or 1), summed to obtain an index representing the level of stress among rural youth under each dimension.

#### Explained variable: Public participation.

The indicator selected to measure public participation was Question H2a: “In the past 2 years, have you participated in the following activities?” There were 12 potential responses to this question, with “10” representing participation in religious activities. China’s Constitution explicitly stipulates that citizens enjoy freedom of religious belief, while emphasizing that the management of religious affairs adheres to the principle of independence and self-governance, actively guiding religions to adapt to socialist society. Against this backdrop, religious activities primarily occur within specific, private spheres. Their organizational forms and purposes fundamentally differ from conventional public participation that take place in the public domain. The latter places greater emphasis on collective actions undertaken by citizens in social and public life to achieve common interests, with objectives and processes characterized by distinct secular and public dimensions. Excluding religious participation helps focus research on forms of public participation that align with core socialist values and directly serve social development and community building. This ensures clear research boundaries and conclusions with greater practical guidance, while also aligning with the state’s advocated direction for grassroots social governance. Therefore “10” was excluded from this study.

The remaining options included discussing political issues, reflecting social issues to the media, providing feedback to government departments, participating in public policy with professional knowledge, public affairs advocacy meetings, publishing opinions on government policies through various channels, attending hearings, petitioning, participating in discussions on major decisions, participating in social welfare activities, and participating in collective rights protection actions, among various forms of public participation. The different types of public participation were regarded as constituting multiple factors of rural youth public participation. Through principal component factor analysis of its internal structure, new factors that can summarize multiple specific indicators were extracted. Each indicator among the new factors was considered as a 0 or 1 variable, summed to obtain an index that represented rural youth public participation behavior within each dimension.

The different types of public participation behaviors were regarded as constituting multiple factors of rural youth public participation. Through principal component factor analysis of its internal structure, new factors that can summarize multiple specific indicators were extracted. Each indicator within these new factors was considered as a 0 or 1 variable, summed to obtain an index that represented rural youth public participation behavior within each dimension.

#### Controlled variables.

Based on existing literature and data, gender, age, education level, and employment status were selected as the control variables. Gender (1 = Male, 2 = Female), age, education level (based on the number of years of compulsory education (12 years) in China.) (1 = uneducated; 2 = Compulsory education, 0–12 years; 3 = Higher education, > 12 years), and employment status (1 = Government institutions, 2 = State-owned/collective enterprises, 3 = Private enterprises/foreign-funded enterprises, 4 = Flexible employment) were chosen as the controlled variables.

### Measurements

The steps of factor analysis are as follows: First, exploratory factor analysis is conducted to assess the reliability and validity of the specific indicators. Second, varimax rotation is applied to the factor loadings to facilitate the comprehensive analysis of the indicators related to life stress. Finally, new factors summarizing multiple specific indicators are extracted. The items within each new factor are treated as binary variables (0,1) and summed to derive indices representing different sources of stress among rural youth.

The criteria for determining the suitability of factor analysis are as follows: 1. The closer the KMO value is to 1, the stronger the partial correlation between variables, indicating better factor analysis results. In practice, a KMO value above 0.6 is considered acceptable; 2. If the test statistic is significant and the corresponding p-value is less than the significance level (typically 0.05), it suggests that there is a significant correlation between variables, making factor analysis appropriate; 3. The higher the Cronbach’s alpha coefficient, the greater the reliability. If the coefficient is less than 0.35, adjustments are necessary.

First, we conducted a reliability and validity test of factor analysis on the life stress of rural youth. The results showed a KMO value of 0.82, a p-value of 0.00 for Bartlett’s test of sphericity, and a Cronbach’s alpha coefficient of 0.71. These results meet the criteria for factor analysis, indicating that factor analysis is appropriate for the life stress indicators among rural youth.

The 13 indicators were summarized into three factors, which were named Family stress, Economic stress, and Social stress, based on the content of the indicators included in each factor and the classification criteria of this study. The communality for each indicator was above 0.5, and the cumulative variance contribution rate of the three factors was 39.344%, meeting the requirements of factor analysis.

Based on these results, the 13 indicators representing different life stress events were re-divided into three dimensions of stress events according to their internal structure: Family stress, Economic stress, and Social stress. The stress indices within each dimension were summed to derive the stress indices of rural youth under different dimensions.

Next, we conducted a reliability and validity test of factor analysis on the public participation behavior of rural youth. The results showed a KMO value of 0.65, a p-value of 0.00 for Bartlett’s test of sphericity, and a Cronbach’s alpha coefficient of 0.48. These results meet the criteria for factor analysis, indicating that factor analysis is appropriate for the public participation indicators among rural youth.

The 11 indicators were summarized into four factors, which were named Expression of attitudes, Decision-making participation, Mutual cooperation, and Collective rights protection, based on the content of the indicators included in each factor and the classification criteria of this study. The communality for each indicator was above 0.5, and the cumulative variance contribution rate of the four factors was 52.783%, meeting the requirements of factor analysis.

Based on these results, the 10 indicators representing different types of public participation behavior were re-divided into four types of public participation behavior according to their internal structure: Expression of attitudes, Decision-making participation, Mutual cooperation, and Collective rights protection. The indices within each dimension were summed to derive the social participation status of rural youth across different types.

## Results

### Descriptive statistical analysis

**Fig 2:**
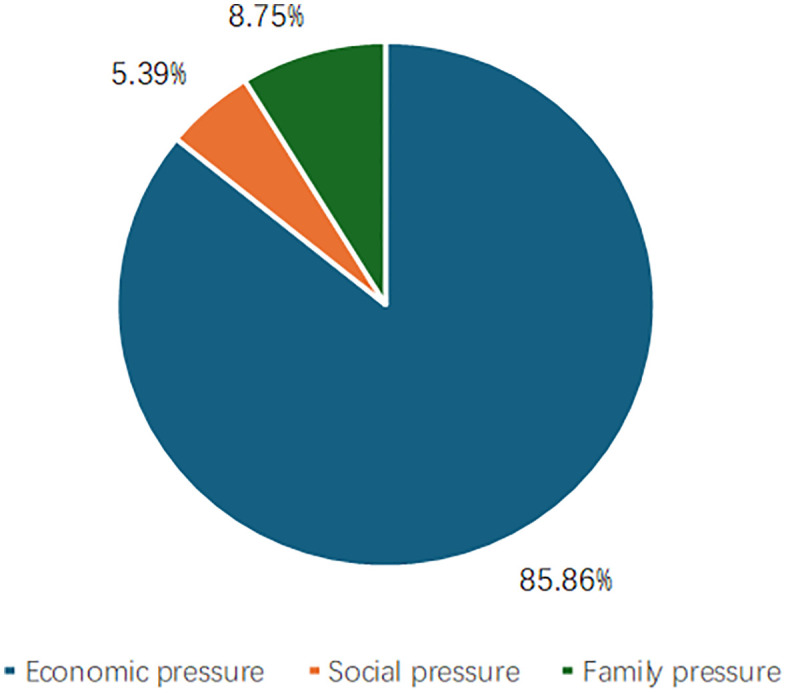
Composition of life stress of rural youth in China.

The greatest stress faced by rural youth is economic stress, while social and family stress was relatively minor (Fig 2). The breakdown of those stressors was as follows: economic stress accounted for 85.86%, with 82.73% of individuals earning below the average income level (69,311.28 yuan). Among such individuals, 61.14% were female, suggesting that female rural youth face heightened economic obstacles such as income inequality and employment disadvantages. Family stress accounted for 8.75%, with 60.56% being youth over 28 years of age, and potentially related to marital status. Unmarried rural youth experience significantly less family stress at only 37.58% with 62.11% of those affected by this stress being female. Such increased stress was likely due to traditional rural beliefs that prioritize women’s roles within the family, thus, increasing family stress for female rural youth. Social stress accounted for 5.39%, with married rural youth accounting for 45.04%. That value was slightly lower than for unmarried rural youth, which, to some extent, reflected the moderating role of family support on social stress.

**Fig 3:**
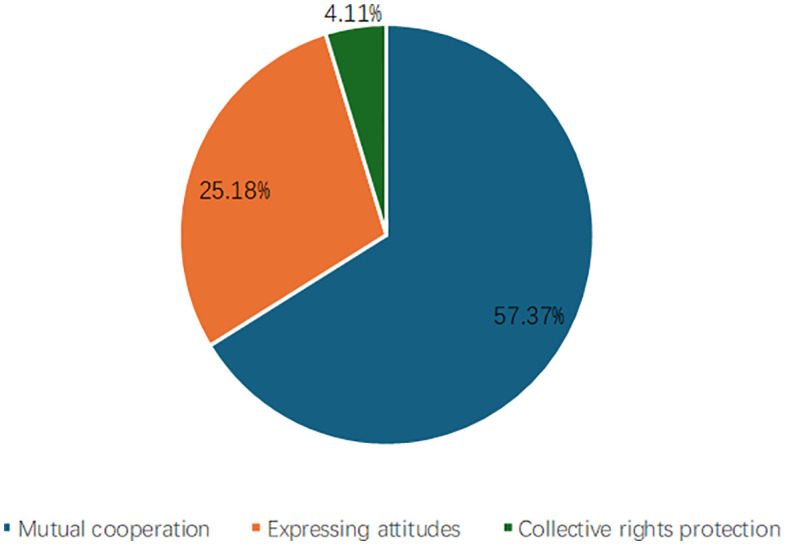
Composition of public participation behaviors of rural youth in China.

The public participation behaviors of rural youth were as Fig 3: over half of rural youth (57.37%) engaged in public participation through mutual cooperation. A significant portion (25.18%) engaged in expressing attitudes. In contrast, the proportion of rural youth engaging in public participation through collective rights protection was minimal, at only 4.11%. Among rural youth engaged in collective rights protection, 68.29% held a bachelor’s degree or higher level of education, while only 2.44% had primary school education or below, thus, indicating a correlation between educational attainment and the modes of public participation. The former two channels of public participation were more prevalent among rural youth, whereas the latter faces certain difficulties and obstacles.

### Base regression analysis

Based on theoretical analysis and hypotheses from previous studies, an empirical examination was conducted on the relationship between life stress among rural youth and their public participation behaviors. A multiple linear regression model (Model 1) was constructed, as represented by Equation (1):


$$\text{E}\left[{\text{Y}}_{\text{i}}\right]=\beta_{\text{0}}+\beta_{\text{1}}{\text{X}}_{\text{1i}}+\beta_{\text{2}}{\text{X}}_{\text{2i}}+\ldots +\beta_{\text{k}}{\text{X}}_{\text{ki}}+\epsilon_{\text{i}}$$ (1)

where β_0_ represents the intercept of the model, β_1_ and β_2_ are coefficients for explanatory variables, β_3_ to β_7_ denote coefficients for control variables, and ε denotes the error term.

The results are shown in Table 1. Economic stress does not significantly affect all forms of public participation behavior. Family stress had no significant impact on attitude expression and decision-making participation, but significantly negatively correlated with cooperative assistance and collective rights defense at levels of 0.1 and 0.05, respectively. Those data indicate that greater family stress reduced the likelihood of rural youth engaging in cooperative assistance and collective rights defense activities.

**Table 1:** Results of multiple linear regression analyses.

Variables	Expression of attitudes	Decision-making participation	Mutual cooperation	Collective rights protection
Economic stress	−0.002 (0.006)	0.003 (0.0034)	−0.003 (0.007)	0.002 (0.002)
Family stress	−0.012 (0.028)	−0.020 (0.018)	−0.059* (0.033)	−0.020** (0.009)
Social stress	0.175*** (0.030)	0.048** (0.019)	0.156*** (0.035)	0.035*** (0.010)
Constant	0.134*** (0.0173)	0.071*** (0.011)	0.347*** (0.020)	0.019*** (0.006)
R^2^	0.021	0.005	0.013	0.011

Note: *** denotes p < 0.001, ** denotes p < 0.01,* denotes p < 0.05. The same as below (unless otherwise stated).

Social stress positively and significantly correlated with all forms of public participation behavior. Thus, as social stress increased, rural youth were more likely to express attitudes, participate in decision-making, engage in cooperative assistance, and participate in collective rights defense. Hypotheses H_1_ and H_2_ were both supported.

The results suggest that when facing life stress, rural youth may prioritize social factors when they attempt to bring about change through public participation, viewing societal challenges as urgent and important. Consequently, they are driven more by societal factors, with economic stress playing a relatively minor role in motivating them in this regard.

Increased social stress encourages individuals to participate more in collective actions, seeking external support and change. Conversely, increased family stress tends to lead individuals towards internal family repairs, seeking support and security within the family, rather than participating in external cooperation or rights defense activities. Such factors thus limit the engagement of rural youth in cooperative assistance and collective rights defense behaviors.

The non-significant relationship between economic stress and public participation among rural youth may be attributed to several contextual factors. Structurally, in many rural Chinese communities, limited institutional channels for participation could diminish the role of individual economic conditions—when participation opportunities are scarce or ineffective, both economically stressed and non-stressed youth may disengage similarly. Culturally, economic pressures may redirect youth focus toward immediate livelihood improvement, such as seeking urban employment or engaging in side occupations, thereby deprioritizing public affairs. Moreover, the nature of public participation in rural settings—often involving informal, kinship-based, or low-cost activities—may not be sensitive to variations in individual economic stress. Methodologically, the measurement of economic stress might not fully capture subjective financial anxiety or localized economic constraints.

### Heterogeneity analysis

Considering the stress that rural youth face and the different ways in which they cope with stress, there may be significant differences in how stressors impact public participation behaviors.

In prior research, we conducted a multiple linear regression analysis of the life stress and public participation behavior among rural youth, examining the impact of three types of life stress on various public participation behaviors. Individual differences play a significant role in coping and the perception of life stress, which may further influence the patterns and extent of public participation.

We further explored how different demographic characteristics affect the life stress of rural youth and their mechanisms of action on public participation behavior. To better understand this complex relationship, we first considered that educational level is typically closely related to an individual’s cognitive style, social participation ability, and strategies for coping with life stress. Rural youth with higher education may possess stronger social resource acquisition capabilities, higher self-efficacy, and more mature emotional regulation mechanisms, which may enable them to adopt more proactive coping strategies when facing life stress, thereby influencing their public participation behavior. Conversely, groups with lower education levels may lack these resources and abilities, leading to differences in the impact mechanisms of life stress on public participation. Secondly, considering that the nature of work of rural youth may directly affect the sources and intensity of their life stress. Physical labor and low-skilled jobs may bring more physical and time stress, while mental labor or more flexible jobs may involve more psychological stress. In addition, different types of jobs may be affected by the working environment, income level, and social status, all of which will affect individuals’ public participation behavior. Therefore, as a demographic characteristic, the nature of work may play an important role in the relationship between life stress and public participation behavior. The following research will conduct a heterogeneity analysis of the relationship between life stress and public participation behavior of rural youth based on two important demographic characteristics that may affect the way life stress affects public participation, namely educational level and nature of work.

Of the total study sample, 1.29% of rural youth were uneducated, 59.30% had compulsory education only, and 39.41% had higher education.

The results are shown in Table 2. For uneducated rural youth, life stress did not significantly influence their public participation behaviors. Uneducated rural youth may adopt different coping strategies than their more educated peers when facing life stress. Uneducated youth may also be more inclined to seek individualized means of stress relief, such as seeking support from family or social networks. Additionally, uneducated rural youth may face greater resource constraints, such as time, money, and social capital. Life stress may also lead such individuals to focus more on fulfilling basic needs, rather than engaging in public participation. Furthermore, lower educational attainment could affect their ability to acquire information and understand complex social participation behaviors, potentially resulting in lower cognitive awareness of public participation and reduced interest and involvement in such behaviors.

**Table 2:** Results of heterogeneity analyses based on education level.

Education level	Variables	Economic stress	Social stress	Family stress	Constant	R^2^
Uneducated	Expression of attitudes	0.095 (1.37)	−0.331 (−1.28)	0.119 (0.50)	−0.126 (−0.51)	0.146
	Decision-making participation	−0.050 (−1.06)	0.040 (0.22)	−0.069 (−0.42)	0.286 (1.69)	0.100
	Mutual cooperation	0.000 (.)	0.000 (.)	0.000 (.)	0.000 (.)	.
	Collective rights protection	0.000 (.)	0.000 (.)	0.000 (.)	0.000 (.)	.
Compulsory education	Expression of attitudes	−0.001 (−0.15)	0.144*** (4.18)	−0.028 (−0.88)	0.104*** (4.87)	0.018
	Decision-making participation	0.003 (0.75)	0.033 (1.54)	0.007 (0.38)	0.056*** (4.27)	0.004
	Mutual cooperation	0.007 (1.13)	0.114*** (3.24)	−0.050 (−1.53)	0.194*** (8.82)	0.014
	Collective rights protection	0.002 (0.98)	0.015* (1.71)	−0.011 (−1.30)	0.009* (1.66)	0.005
Higher education	Expression of attitudes	0.002 (0.18)	0.278*** (4.96)	0.048 (0.85)	0.156*** (5.36)	0.041
	Decision-making participation	0.010 (1.33)	0.085** (2.31)	−0.070* (−1.89)	0.077*** (4.01)	0.015
	Mutual cooperation	0.011 (0.77)	0.278*** (4.03)	0.033 (0.47)	0.497*** (13.80)	0.029
	Collective rights protection	0.008* (1.75)	0.080*** (3.63)	−0.033 (−1.49)	0.023** (2.00)	0.028

For rural youth with compulsory education, their expression of attitudes, mutual cooperation and collective rights were significantly positively influenced by social stress. In terms of attitudinal expression, social expectations can shape their positive attitudes, and social comparisons can stimulate their upward mobility; in terms of mutual cooperation, social stress can stimulate their motivation to cooperate, and common goals under social stress can promote their sense of cooperation; and in terms of collective rights, social stress can enhance the awareness and cognition of rights. Rural youth with compulsory education are more likely to be motivated to participate in society under social stress for the following reasons: 1) cognitive enhancement: compulsory education enhances their ability to understand social phenomena and cope with stress; 2) internalization of values: education enables them to internalize social norms and enhance their sense of social responsibility and willingness to participate; 3) enhancement of adaptive capacity: the skills they cultivate, such as communication and collaboration, help them better adapt to their social environments; 4) awakening of their rights awareness: education awakens a sense of self and rights, prompting them to actively defend their own rights and interests; 5) expansion of resources: the social connections established provide them with information and support, and help them to participate in society.

For rural youth with a higher education, their collective rights protection was significantly positively influenced by economic stress. All types of public participation were positively influenced by social stress, while family stress negatively influenced their decision-making participation. Family stress inhibits decision-making participation among highly educated rural youth, possibly because of familial expectations, opposition, or other forms of stress that restrict decision-making processes. Individuals with higher levels of education may have already achieved a balance in terms of societal expectations and prioritize fulfilling familial expectations. Therefore, family stress could serve as a barrier to their participation in decision-making activities. Thus, hypothesis H_3_ was confirmed.

The results are shown in Table 3. For rural youth engaged in agricultural work, their expression of attitudes was positively influenced by social stress and negatively affected by family stress. The nature of agricultural work is somewhat unique, often involving frequent and close contact with family members. Thus, agricultural youth may rely more on family economic support, being more sensitive to family stress. Economic stress directly impacts the daily lives and agricultural production of rural youth working in the agriculture industry, potentially resulting in stronger negative effects on their attitude expression. This dependence may also be reciprocal, as rural youth directly face economic stress related to family expectations, financial conditions, and uncertainties in agricultural production. Such family stress significantly influences their attitude expression, making them more cautious or conservative and less willing to express radical or uncertain viewpoints.

**Table 3:** Results of heterogeneity analyses based on works.

	Variables	Economic stress	Social stress	Family stress	Constant	R^2^
Agricultural work	Expression of attitudes	0.003 (0.41)	0.236*** (6.11)	−0.063* (−1.70)	0.104*** (4.71)	0.053
	Decision-making participation	0.003 (0.56)	0.017 (0.70)	0.014 (0.62)	0.045*** (3.26)	0.003
	Mutual cooperation	−0.011 (−1.01)	0.209*** (3.74)	−0.135** (−2.50)	0.437*** (13.59)	0.027
	Collective rights protection	−0.000 (−0.03)	0.031** (2.00)	−0.026* (−1.76)	0.027*** (3.13)	0.009
Government or public sector organizations	Expression of attitudes	−0.006 (0.010)	0.174*** (0.054)	0.009 (0.051)	0.152*** (0.031)	0.017
	Decision-making participation	0.002 (0.006)	0.076** (0.032)	−0.026 (0.031)	0.083*** (0.018)	0.010
	Mutual cooperation	0.006 (0.010)	0.126** (0.054)	0.041 (0.051)	0.287*** (0.031)	0.013
	Collective rights protection	0.005* (0.003)	0.058*** (0.015)	−0.020 (0.015)	0.007 (0.009)	0.031
State-owned or collective enterprises	Expression of attitudes	−0.003 (−0.25)	0.034 (0.46)	0.052 (0.79)	0.157*** (3.25)	0.003
	Decision-making participation	0.000 (0.010)	0.065 (1.24)	−0.087* (−1.83)	0.126*** (3.60)	0.016
	Mutual cooperation	0.004 (0.32)	0.100 (1.43)	−0.084 (−1.32)	0.228*** (4.90)	0.013
	Collective rights protection	0.001 (0.25)	0.000 (0.00)	−0.011 (−0.58)	0.022 (1.52)	0.001

Conversely, in rural society, social stress may involve societal expectations and village norms, while family stress may be linked to traditional values and agricultural production. The interaction between these factors may significantly negatively impact attitude expression among purely agricultural rural youth. Moreover, in rural areas, traditional beliefs may deem that the expression of radical or dissenting viewpoints in society are inappropriate, emphasizing instead the need to portray family responsibilities and traditional values. Such values could lead to the negative effects of family stress on attitude expression.

For rural youth working in government or public sector organizations, social and economic stress significantly affects their collective rights protection behaviors. Given the specific economic stress in those work environments, including salary levels, welfare policies, and promotional opportunities, rural youth may more easily connect economic stress with their work environment, thus, engaging in collective rights protection behaviors when facing economic challenges. In such organizational environments, characterized by specific structures and work cultures, rural youth may seek support from colleagues and the organization, fostering unified collective rights protection behaviors. Economic stress is also a focal point of concern, which encourages youth to actively participate in collective rights protection. Furthermore, government and public sector organizations typically have clear organizational structures and cultures. In such environments, when rural youth experience economic stress, they may prefer to express dissatisfaction with internal economic systems through collective rights protection. Some government and public sector organizations may face unique economic challenges, such as budget constraints and fiscal stress, which further influence economic stress on collective rights protection behaviors.

For rural youth employed in state-owned or collective enterprises, family stress was significantly negatively correlated with their decision-making participation behaviors, while social stress no longer remained a significant factor. First, considering the organizational structure, state-owned and collective enterprises are subject to an increasing number of internal regulations and controls from government or organizational bodies. In such organizations, individual decisions may be influenced by internal rules and organizational culture, with relatively little influence from social stress. Second, work in those organizations may involve clearer professional responsibilities and organizational missions, causing individuals to prioritize internal organizational expectations and requirements over external social stress. Stricter organizational cultures and management systems may limit employee public participation. Social stress may therefore not significantly influence public participation behaviors in this context, compared to other organization types where social stress may have a more pronounced impact. Additionally, rural families typically have higher expectations for their children, especially when working in stable environments such as state-owned or collective enterprises. Families may desire stable income and benefits from jobs, aiming to improve family economic conditions and fulfill family responsibilities. Thus, family stress could significantly influence decision-making participation among those rural youth.

Rural families generally emphasize family continuity, stability, and mutual assistance, encouraging rural youth to prefer employment in stable environments, such as state-owned or collective enterprises, to obtain job stability and economic security, and thereby reducing stress on the family. This stability-seeking mindset may reduce public participation among rural youth. Finally, rural youth choosing employment in stable environments may face greater stress due to the need to support family members and pay education expenses. Such economic stress may compel rural youth to prioritize economic stability and be less willing to participate in public affairs.

#### Summary Table: Patterns of life stress impact on public participation (by group).

**Table:** 

Public Participation Behavior	Education level	Works
Expression of attitudes	Driven by Social Stress: Significant positive influence in both compulsory and higher education groups.	Driven by Social Stress, Inhibited by Family Stress: Among the agricultural work group, social stress is a positive driver, while family stress exerts a significant negative influence.
Decision-making participation	Inhibited by Family Stress: A significant negative influence from family stress is found only in the higher education group.	Inhibited by Family Stress: Family stress is the primary inhibiting factor for youth in state-owned or collective enterprises.
Mutual cooperation	Driven by Social Stress: Significant positive influence in both compulsory and higher education groups.	Driven by Social Stress, Inhibited by Family Stress: Among the agricultural work group, social stress is a positive driver, while family stress exerts a significant negative influence.
Collective rights protection	Driven by Multiple Stressors: • Compulsory Education: Driven by social stress. • Higher Education: Driven by both economic stress and social stress.	Driven by Economic & Social Stress: Among youth in government/public sectors, both economic and social stress show significant positive influences.

### Robustness check

The results of multiple linear regression analyses indicated a strong correlation between life stress and the public participation behavior of youth. Whether a causal relationship truly exists between the two factors must be determined after addressing endogeneity issues. Endogeneity could arise from two aspects: first, reverse causality, where rural youth engaging in public participation may encounter difficulties that subsequently alter their life stress conditions. Second, omitted variable bias. Individual characteristics of rural youth that are unobserved may also influence their public participation behavior. To address these factors, alternative model specifications and propensity score matching methods will be employed in future studies to perform a robust reassessment of the study’s conclusions.

#### Replace the model.

Under large sample conditions, Ordinary Least Squares (OLS) and Probit models are essentially equivalent. Therefore, we re-estimated the model using the OProbit approach and present the results in Table 4. Economic stress had a positive impact on public participation behavior, which was significant at the 5% significance level. Additionally, social stress also positively influenced public participation behavior, which was significant at the 1% level. Thus, the positive effects of economic and social stress on public participation behavior persisted under different model assessment.

**Table 4:** Robustness check (Oprobit).

	Coefficient	Standard Error	z	p	Confidence interval	
Economic stress	0.033	0.016	2.05	0.040	0.001	0.064
Family stress	−0.106	0.079	−1.35	0.178	−0.260	0.048
Social Stress	0.306	0.082	3.73	p < 0.001	0.145	0.466
Gender	−0.180	0.681	−2.63	0.0008	−0.313	−0.046
Age	−0.036	0.072	−4.97	p < 0.001	−0.049	−0.021
Education	0.509	0.060	8.48	p < 0.001	0.391	0.626
Work	0.062	0.051	1.20	0.229	−0.039	0.162
Cons.	−0.382	0.273	−1.40	0.162	−0.917	0.154

#### Propensity score matching.

Propensity score (PS) is a function of multiple covariates used to address the issue of uneven covariate distributions between groups in observational studies. Propensity score matching (PSM) is a statistical method used in intervention effect analysis with non-experimental or observational data, aiming to estimate treatment effects. The treatment and control groups were divided based on the presence or absence of life stress variables: the treatment group included rural youth experiencing life stress (economic stress, family stress, or social stress), while the control group consisted of rural youth without life stress.

The use of PSM is to mitigate selection bias in observational data. In non-randomized experimental designs, systematic differences may exist between treatment and control groups in baseline characteristics, which can affect the estimation of treatment effects. PSM addresses this by matching members of the treatment and control groups who have similar propensity scores — the conditional probability of receiving treatment given observed covariates. This approach simulates conditions of a randomized experiment to reduce bias between groups.

**Fig 4:**
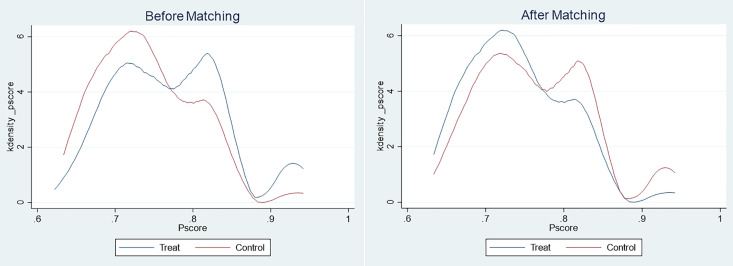
Kernel density estimate.

By plotting kernel density graphs (Fig 4), it was evident that there were significant differences between the control and treatment groups before matching, which narrowed after matching. Nearest neighbor, radius, and kernel matching methods all indicated a positive impact of the treatment group over the control group, with the post-matching Average Treatment Effect on the Treated (ATT) values consistently higher than the unmatched results, thus, indicating an effective reduction of inter-group bias. According to Table 5, among the three matching methods, radius matching exhibited the highest ATT value, suggesting it provides the strongest estimation of treatment effects under those conditions. Moreover, the standard deviations and t-values demonstrated statistical stability and significance of the results. All ATT estimates had t-values greater than 2, indicating statistical significance and enhancing the credibility of the findings. Thus, it can be inferred that there are behavioral differences in the forms of public participation between the treatment group (rural youth experiencing life stress) and the control group (rural youth without life stress).

**Table 5:** PSM results.

Matching method		Treatment group	Control group	ATT	Standard error	T
k-Nearest Neighbors Matching (1:3)	Unmatched	0.658	0.528	0.130	0.057	2.290
	ATT	0.657	0.425	0.232	0.064	3.640
	ATU	0.528	0.687	0.159	–	–
Radius Matching (0.01)	Unmatched	0.658	0.528	0.130	0.057	2.290
	ATT	0.665	0.428	0.237	0.062	3.800
	ATU	0.528	0.687	0.159	–	–
Kernel Matching	Unmatched	0.658	0.528	0.130	0.057	2.290
	ATT	0.657	0.448	0.209	0.057	3.670
	ATU	0.528	0.741	0.212	–	–

Building on previous specific conclusions, economic stress and family stress do not significantly influence attitude expression and decision-making participation, suggesting these stress factors are insufficient to drive or inhibit rural youth activity in those areas. However, social stress significantly affects both forms of participation, positively correlating with them, and thus, indicating that increased social stress may motivate rural youth to express their attitudes more or participate in decision-making processes. Conversely, family stress significantly and negatively influences cooperative assistance and collective rights defense, suggesting that rural youth are less likely to engage in these actions under greater family stress. In contrast, an increase in social stress correlates significantly and positively with increased participation in these public forms.

In summary, PSM analysis revealed how different dimensions of life stress affect the social participation behavior of rural youth. In particular, social stress acts as an incentive mechanism, encouraging youth to be more active in public participation. These findings are crucial to understand social dynamics and the behavioral patterns of youth in rural areas.

## Strengths and limitations

### Strengths

First, this study extends the stress process theory from psychology and health to demography, particularly in analyzing the life stress of rural youth and its impact on public participation behavior, which is relatively rare in the existing literature.

Second, this study not only examines the direct impact of life stress on public participation behavior but also explores the specific impacts of different sources of life stress, such as economic, family, and social stress, on public participation behavior and the differences in these impacts among different groups.

Finally, this study integrates theories and methods from demography, sociology, and psychology, forming an innovative interdisciplinary research perspective.

### Limitations

This study systematically analyzes the impact of life stress on public participation behavior among rural youth, but there are still several limitations due to questionnaire design, variable measurement, data availability, and literature coverage.

First, the measurement of core variables requires further refinement. Although life stress and public participation were constructed based on items from the CSS2021 questionnaire, the binary-sum index used for participation may overlook variations in frequency and intensity. This simplification could affect the interpretation of the relationship between stress and participation, as different thresholds or forms of engagement might respond differently to life stressors.

Second, the study did not fully account for potential latent variables and individual adaptation mechanisms. Respondents may employ diverse coping strategies when facing life stress, and such heterogeneity could mask underlying patterns in how stress influences participation. Moreover, other unmeasured factors—such as personal emotions, informal social networks, and local cultural norms—may shape both stress perception and participatory behavior, yet these were not explicitly included in the current model.

Third, while the study aimed to contextualize rural youth participation within broader scholarly discourse, the literature review could be expanded. In particular, recent studies focusing on digital participation and comparative rural civic engagement in international settings are not sufficiently incorporated, partly due to the limited availability of such literature. This constraint may affect the generalizability and contemporary relevance of the findings.

Finally, although all figures and tables were designed to support key findings, future revisions should ensure that each is explicitly discussed and tied to specific results to enhance clarity and interpretive depth.

## Discussion and conclusions

Since the 18th National Congress of the Communist Party of China, the Party has consistently focused on ensuring national long-term stability and people’s well-being, continuously improving the social governance system, and achieving significant milestones in modernizing social governance, thus, continuing long-term social stability. Rural youth, as a vital demographic, and beneficiaries, participants, and contributors to public social governance, play an indispensable role in the improvement of the social governance system. To explore ways to enhance the level of public participation of rural youth, this study used data from the CSS 2021 to analyze the impact of life stress on the public participation behaviors of this demographic, followed by further heterogeneous analysis.

### Conclusion

The conclusions of the study are as follows: First, life stress significantly influences the public participation behavior of rural youth. Second, social stress significantly affects various forms of public participation among rural youth, and shows a positive correlation. Additionally, family stress has a significant negative impact on the participation of rural youth in mutual assistance and collective rights protection. Economic stress, however, does not significantly influence the public participation behavior of rural youth. Third, through heterogeneous analyses, it was found that economic stress had a significant positive impact on collective rights protection behavior among rural youth, with higher levels of education and employment in government institutions, after controlling for sample characteristics, including education level and employment type.

### Discussion

A key finding of the study was the significant negative impact of family stress on the public participation behavior of rural youth. From the perspective of social psychology, this can be understood in terms of family dynamics and individual autonomy. In rural areas, the family often plays a central role in decision-making, with social and economic expectations exerting considerable influence on the individual. Family stress—whether related to financial responsibilities, traditional expectations, or caregiving roles—may limit the autonomy of rural youth, making it more difficult for them to participate in broader social and political activities. This may be particularly pronounced in rural areas, where traditional family roles are more deeply entrenched.

Psychological Strain is one mechanism through which family stress affects public participation. Such stress can cause significant psychological strain, leading to heightened anxiety and reduced emotional bandwidth for engaging in public activities. When rural youth are preoccupied with family-related stressors—such as caregiving or managing household responsibilities—they may feel too overwhelmed or emotionally drained to connect with broader community or public affairs.

Beyond individual psychological effects, these pressures are often compounded by Social Conformity. In many rural communities, youth are socialized to view their primary obligation as being to the family, which can suppress independent action or engagement in public matters. This tendency is reinforced by social norms that emphasize conformity, making it harder for young individuals to prioritize public participation over familial expectations.

Moreover, the issue is further intensified by a Lack of Social Capital. Families in rural areas may not be well-integrated into broader social networks and often have fewer resources to participate in collective actions. This lack of social and relational capital not only limits opportunities for public involvement but also reduces the likelihood that rural youth will seek external support, thereby reinforcing the negative impact of family stress.

This dynamic is deeply embedded in the broader Rural Social Structure, which often exacerbates the relationship between family stress and public participation. In many rural communities, intergenerational authority structures place a strong emphasis on the younger generation’s responsibility to support the family—economically, socially, and emotionally. These responsibilities inevitably reduce the time and energy available for public engagement.

Finally, these structural and normative pressures are frequently sustained by Dependency on Family Income. Rural families often rely on a small number of income earners and maintain strictly delineated family roles. When facing economic stress at home, rural youth are often expected to work or contribute directly to the household’s well-being, further constraining their ability to participate in activities beyond their immediate familial duties.

Family stress stem from multiple dimensions including economic responsibilities, generational expectations, caregiving burdens, and traditional role norms. Primarily functioning as an external stressor, they directly impact the psychological state of rural youth. This stress is not a static external variable but a persistent psychological burden, readily triggering a series of negative emotions such as anxiety, depression, and helplessness. These negative emotions not only deplete an individual’s psychological energy but also significantly impair cognitive functions and decision-making patterns.

**Narrowed Attention.** Family stress compel rural youth to continuously direct their limited attention resources toward internal household matters, thereby diminishing their focus on community public issues and their ability to absorb relevant information;

**Heightened Risk Aversion.** Under the influence of anxiety, individuals become more sensitive and conservative in assessing potential risks. Public participation activities like collective advocacy, due to their uncertain outcomes, are often perceived by rural youth as high-risk activities, leading to active avoidance;

**Reduced self-efficacy.** Prolonged stress and negative emotions erode confidence in one’s capabilities, fostering the belief that even participation cannot change the status quo, thereby diminishing intrinsic motivation for engaging in public affairs.

Heavy family burdens trigger psychological tension. If this tension cannot be buffered by effective social support or personal resources, it solidifies into a persistent negative emotional state. These emotional states further distort or reinforce specific cognitive biases. The avoidance of collective rights advocacy is not merely a matter of time and energy allocation, but rather the combined effect of anxiety-driven risk avoidance and motivation loss caused by depression: cognitively, family responsibilities are internalized as primary obligations, while public participation is constructed as a secondary option or even an unnecessary risk. This cognitive hierarchy becomes more entrenched under heightened emotional stress. Thus, the emotional mediation mechanism explains why rural youth burdened by family stress choose adaptive abandonment despite potential gains from public engagement—not as a simple rational calculation, but as a behavioral outcome stemming from emotional burden and cognitive resource depletion.

Ultimately, family stress exerts its strongest inhibitory effect on collective rights advocacy—activities requiring perceived risk-taking and sustained mental investment—primarily by inducing anxiety and competing for cognitive resources. In contrast, its negative impact is relatively weaker on lower-risk activities like mutual aid or expressing opinions, which may even stimulate specific forms of participation driven by the need for mutual support.

In summary, the core mechanism through which family stress influences rural youth’s public participation lies in triggering negative emotional mediating processes centered on anxiety and depression. These emotional states subsequently lead to depleted cognitive resources, amplified risk perception, and diminished self-efficacy. This psychological chain ultimately results in avoidance of public participation, particularly high-investment, high-risk engagement behaviors. Social support and personal resources can act as buffers at different points along this chain, altering the intensity of emotional responses and the direction of cognitive evaluations.

Based on the key statistical findings of this study, the following evidence-based recommendations are proposed to enhance public participation among rural youth in China.

1. Leverage social stress for mobilization.

Design community programs that channel social stress into constructive participation. Instead of merely reducing social stress, create targeted mobilization campaigns that convert stress-driven awareness into active civic involvement. Firstly, develop “stress-aware” participatory platforms where youth can address sources of social stress through structured dialogue and collective action. Secondly, train community leaders to identify and recruit youth experiencing high social stress, offering them roles in public initiatives that align with their concerns.

1. Alleviate family and economic stressors to enable participation.

Implement integrated support programs that reduce practical barriers to engagement. Firstly, introduce family therapy and financial literacy workshops in rural areas to help youth manage domestic and economic pressures. Secondly, offer conditional cash transfers or small grants for youth who participate in public activities, mitigating opportunity costs.

1. Strengthen mental health and stress management systems.

Institutionalize mental health services within rural communities, with a focus on stress coping and resilience building. Firstly, integrate mental health check-ups into existing rural healthcare services. Secondly, offer group counseling and stress-management workshops tailored to youth, linking these sessions to public participation opportunities.

1. Enhance civic education and participation literacy.

Embed participatory skills and civic rights education into rural schooling and adult education programs. Firstly, revise rural school curricula to include project-based learning in community issues. Secondly, use social media to disseminate simplified information about public affairs and legal rights.

1. Build differentiated support for key subgroups.

Tailor programs to specific youth segments. Firstly, create leadership tracks for highly educated rural youth in local governance. Secondly, offer vocational and public-speaking training for less-educated youth to build confidence and skills.

## Supporting information

S1 File (XLSX)
